# Study protocol for a pragmatic trial of the Consult for Addiction Treatment and Care in Hospitals (CATCH) model for engaging patients in opioid use disorder treatment

**DOI:** 10.1186/s13722-019-0135-7

**Published:** 2019-02-19

**Authors:** Jennifer McNeely, Andrea B. Troxel, Hillary V. Kunins, Donna Shelley, Joshua D. Lee, Alexander Walley, Zoe M. Weinstein, John Billings, Nichola J. Davis, Roopa Kalyanaraman Marcello, Bruce R. Schackman, Charles Barron, Luke Bergmann

**Affiliations:** 1 Department of Population Health, Section on Alcohol, Tobacco and Drug Use, NYU School of Medicine, 180 Madison Avenue, 17th floor, New York, NY 10016 USA; 2 Department of Medicine, Division of General Internal Medicine and Clinical Innovation, NYU School of Medicine, 462 1st Avenue, New York, NY 10016 USA; 3 NYC Department of Health and Mental Hygiene, Bureau of Alcohol and Drug Use Prevention Care and Treatment, 42-09 28th Street, Room CN14, Queens, NY 11101 USA; 4 Clinical Addiction Research and Education Unit, Boston University School of Medicine, 801 Massachusetts Ave., 2nd Foor, Boston, MA 02118 USA; 5 Wagner School of Health Policy and Public Service, New York University, 295 Lafayette Street, New York, NY 10012 USA; 6 Office of Population Health, NYC Health and Hospitals, 199 Water Street, New York, NY 10038 USA; 7 Department of Healthcare Policy and Research, Weill Cornell Medical College, 425 E. 61st St., Ste 301, New York, NY 10065 USA; 8 Department of Population Health, Division of Biostatistics, NYU School of Medicine, 180 Madison Avenue, 5th floor, New York, NY 10016 USA; 9 Office of Behavioral Health, NYC Health + Hospitals, 125 Worth St, New York, NY 10013 USA

**Keywords:** Protocol, Addiction, Consult service, Substance use disorder, Opioid use disorder, Hospitalization, (MeSH term) Inpatients, (MeSH term) Opioid substitution treatment, (MeSH term) Consultation

## Abstract

**Background:**

Treatment for opioid use disorder (OUD) is highly effective, yet it remains dramatically underutilized. Individuals with OUD have disproportionately high rates of hospitalization and low rates of addiction treatment. Hospital-based addiction consult services offer a potential solution by using multidisciplinary teams to evaluate patients, initiate medication for addiction treatment (MAT) in the hospital, and connect patients to post-discharge care. We are studying the effectiveness of an addiction consult model [Consult for Addiction Treatment and Care in Hospitals (CATCH)] as a strategy for engaging patients with OUD in treatment as the program rolls out in the largest municipal hospital system in the US. The primary aim is to evaluate the effectiveness of CATCH in increasing post-discharge initiation and engagement in MAT. Secondary aims are to assess treatment retention, frequency of acute care utilization and overdose deaths and their associated costs, and implementation outcomes.

**Methods:**

A pragmatic trial at six hospitals, conducted in collaboration with the municipal hospital system and department of health, will be implemented to study the CATCH intervention. Guided by the RE-AIM evaluation framework, this hybrid effectiveness-implementation study (Type 1) focuses primarily on effectiveness and also measures implementation outcomes to inform the intervention’s adoption and sustainability. A stepped-wedge cluster randomized trial design will determine the impact of CATCH on treatment outcomes in comparison to usual care for a control period, followed by a 12-month intervention period and a 6- to 18-month maintenance period at each hospital. A mixed methods approach will primarily utilize administrative data to measure outcomes, while interviews and focus groups with staff and patients will provide additional information on implementation fidelity and barriers to delivering MAT to patients with OUD.

**Discussion:**

Because of their great potential to reduce the negative health and economic consequences of untreated OUD, addiction consult models are proliferating in response to the opioid epidemic, despite the absence of a strong evidence base. This study will provide the first known rigorous evaluation of an addiction consult model in a large multi-site trial and promises to generate knowledge that can rapidly transform practice and inform the potential for widespread dissemination of these services.

*Trial registration*: NCT03611335

## Background

The US is in the midst of an unprecedented opioid crisis, and the increasing prevalence of opioid use disorders (OUD) and rates of overdose deaths are the nation’s most urgent medical and public health issue [1, 2]. Despite efforts to decrease the inappropriate prescribing of opioids, the number of patients with OUD has risen annually for the past decade [3, 4]. In 2017, the US suffered over 72,000 drug overdose (OD) deaths, more than two-thirds of which were opioid-related [2]. Driven by increases in heroin and synthetic opioid deaths, opioid-related overdose deaths increased by 71% from 2014 to 2017 [5]. Mirroring national trends, the overdose death rate in New York City (NYC) in 2017 was 1221 per 100,000, and 80% of all OD deaths were opioid-related [6, 7].

Motivated by the high morbidity and mortality of OUD as well as the high costs of medical care in this population, public health authorities and health systems throughout the US are looking for effective ways of engaging individuals with OUD in treatment [8–11]. In NYC, the Mayor’s Office launched in March 2017 the ambitious HealingNYC initiative, committing $38 million each year to programs aimed at reducing opioid overdose deaths [7]. This initiative leverages the resources of the city’s public hospital system (NYC Health + Hospitals (H + H)) and the Department of Health and Mental Hygiene (DOHMH) to increase access to OUD treatment. NYC already benefits from a large methadone maintenance treatment system, which is a legacy of the last heroin epidemic of the 1970s. Under HealingNYC, the city plans to increase the number of patients receiving medication for OUD by 20,000 (from the current 38,000) by the year 2022. A key component of this initiative is the introduction of addiction consult services, through the ‘Consult for Addiction Treatment and Care in Hospitals (CATCH)’ program, which is being introduced into six H + H hospitals.

Addiction consult services provide assessment and treatment of substance use disorders in patients who are hospitalized for any condition. Hospitals have a high concentration of individuals with opioid use disorder [12, 13], most of whom receive no OUD treatment [14]. In keeping with the typical organization of inpatient subspecialty care, the addiction consult service provides expert evaluation, diagnosis, and treatment for patients with substance use disorders. A core function of addiction consult services is initiating medication for addiction treatment (MAT) while patients are hospitalized, and linking patients to ongoing maintenance pharmacotherapy as part of the discharge plan. Inpatient addiction consult teams currently exist in multiple health systems, and have been observed to be well accepted by patients and by medical staff who often feel unprepared to treat addiction [8–11]. However, the evidence demonstrating the effectiveness of these services for improving treatment and health outcomes for patients with OUD is limited.

There is reason for optimism about the ability of addiction consult services to improve outcomes by increasing the delivery of MAT. MAT is highly effective at reducing the morbidity, mortality, and costs of OUD [15–17], yet nationwide, less than half of individuals with OUD receive adequate treatment [18, 19]. NYC has no waiting list for methadone maintenance treatment, and HealingNYC funds are supporting the expansion of primary care buprenorphine treatment into 26 new clinical sites, including 3 clinics in the H + H system. Yet research has shown that even when treatment is available it takes considerable effort to engage individuals in care [20–24].

A growing body of evidence indicates that treating OUD during hospitalization can lead to initiation and engagement in MAT after discharge. A randomized controlled trial by Liebschutz et al. [25] tested the efficacy of hospital-initiated buprenorphine treatment versus detoxification and found that the buprenorphine treatment group had significantly higher treatment initiation and lower rates of opioid use following discharge. A number of observational studies have similarly shown increases in post-discharge treatment initiation [10, 26–28], and decreases in addiction severity and opioid use [9] among patients with OUD who received MAT from an addiction consult service during hospitalization. However, prior studies have not evaluated the effectiveness of addiction consult services in comparison to a control condition for hospital inpatients when the service is implemented at scale by clinical staff and integrated into regular medical care.

### Study objectives and specific aims

The overarching objective of our study is to evaluate the effectiveness of CATCH as a strategy for engaging patients with OUD in MAT. A pragmatic trial at 6 hospitals, conducted in collaboration with the New York City public hospital system (H + H) and Department of Health and Mental Hygiene (DOHMH) will study the CATCH intervention in real-world settings and at scale. Our hybrid effectiveness-implementation study focuses primarily on effectiveness, but also measures implementation outcomes to inform the intervention’s adoption and sustainability. A rigorous stepped-wedge cluster randomized trial design determines the impact of CATCH on opioid treatment outcomes in comparison to usual care for a control period, followed by a 12-month intervention period and a maintenance period, and utilizes existing administrative data to evaluate outcomes. Aim 1 (primary aim) is to evaluate the effectiveness of CATCH in increasing post-discharge *initiation* and *engagement* in MAT, defined respectively as receiving outpatient MAT within 14 days of discharge, and having at least 2 additional MAT visits in the first month. Aim 2 is to assess the effectiveness of CATCH for increasing treatment *retention*, defined as continuous receipt of MAT for 6 months. Aim 3 is to compare the frequency of *acute care utilization* and *overdose deaths*, and their associated costs, among patients with OUD who are hospitalized during the CATCH period versus usual care. Aim 4 is to evaluate implementation outcomes at CATCH hospitals using a mixed-methods approach to assess the intervention’s *Reach* (proportion of eligible patients reached); *Adoption* (utilization by medical staff); and *Implementation fidelity* (barriers and facilitators of delivering high-quality care).

## Methods/design

### Study setting

H + H is the largest municipal health care system and the second largest public health care system (following the VA) in the US. H + H provides essential medical services to 1.1 million individuals every year at more than 70 facilities across NYC. It is the primary source of medical care for low-income New Yorkers, and its patient population reflects the diversity of NYC. Over 60% of opioid-related ED visits in NYC are to an H + H facility, and H + H is a major provider of addiction treatment services, with 11 outpatient treatment programs, four opioid treatment programs, and seven licensed detox programs. CATCH teams will be launched in six of the system’s largest acute care hospitals.

### Evaluation framework: RE-AIM

**Fig. 1:**
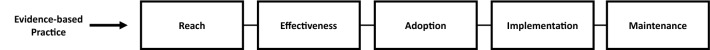
RE-AIM evaluation framework

Our study compares the impact of the CATCH intervention versus treatment as usual (TAU) on OUD treatment initiation, engagement, and retention while assessing its adoption and implementation in practice. The assessment plan is organized by the ‘Reach, Effectiveness, Adoption, Implementation, and Maintenance (RE-AIM)’ framework, one of the most widely applied frameworks for evaluation of health behavior change programs (see Fig. 1) [29]. RE-AIM is appropriate for a pragmatic trial because it offers a framework for measuring the impact of an intervention *as delivered in real world environments*. Assessment of the five domains of RE-AIM provides an evaluation of a program’s potential public health impact. In our study, *Reach* is the proportion of patients who receive CATCH services*. Effectiveness* is the rate of OUD treatment initiation and engagement (primary outcome). *Adoption* is defined as the utilization of the CATCH teams by clinical staff, and measured by the rate of referrals of patients with OUD; and *Implementation fidelity* is the ability of the CATCH teams to identify and reach their target population, and deliver high-quality care. We have limited ability to study *Maintenance* in the time-limited context of this study, but the stepped-wedge design will allow us to continue measuring *Reach* and *Effectiveness* for an additional 6–18 months following the intervention period at each hospital.

### Study design

Our study is a pragmatic trial using a hybrid Type 1 effectiveness-implementation design. Distinct from pure effectiveness trials, hybrid studies aim to also answer the question, “What are the barriers and facilitators to ‘real-world’ implementation of the intervention?” [30, 31] Hybrid Type 1 designs are appropriate when there is strong face validity and at least indirect evidence supporting the intervention, and it is associated with minimal risk to patients. As a pragmatic trial, this study is conducted in partnership with a large public health care delivery system and conducted in real-world care delivery settings that include diverse and representative patient populations, and it measures outcomes that are meaningful to decision makers [32–34].

**Fig. 2:**
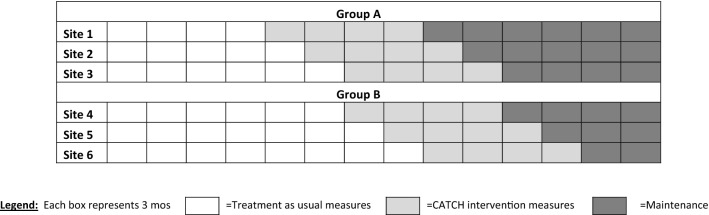
Stepped-wedge design with 6 sites

The stepped-wedge randomized trial design was selected because the H + H system was not amenable to parallel randomization, and intended to implement CATCH via a sequential rollout such that all hospitals would enter into the intervention condition by the end of 2019. Each of the 6 hospitals represents a cluster, and will be evaluated pre-and post-implementation of the CATCH intervention. As is standard in a stepped-wedge trial, each hospital will receive the intervention, but they will implement it at a start date that is randomly assigned, which improves the ability to make causal inferences [35–37]. CATCH is a new program that requires substantial preparation, including hiring new staff, adapting health information technology systems to support the program, and developing new outpatient clinical resources and referral networks. Because the stepped-wedge design requires all hospitals to be at a common level of readiness prior to randomization, to increase feasibility we will randomize the six hospitals in 2 groups: Group A (3 hospitals) will be randomized in late 2018 and Group B (3 hospitals) in early 2019. Within each group, the start times are separated by 3 months (Fig. 2).

Each hospital has a 12-month TAU observation period followed by the 12-month CATCH intervention period. Study outcomes are measured cross-sectionally at 6 and 12 months following introduction of CATCH at each hospital, but data collection will continue for up to 18 months after the intervention period, thereby providing initial information on the maintenance of outcomes. The primary outcome is the rate of MAT initiation and engagement following hospital discharge among patients with OUD, as measured by Medicaid claims and encounter data. We will report outcomes data consistent with CONSORT 2010 guidelines for cluster-randomized trials [38] and modified for a stepped-wedge design as recommended by Hemming [35].

## Description of study conditions

Hospitals in the TAU period provide care for patients with OUD using existing staff and resources. Each hospital has a psychiatry consult service that may treat patients with OUD. Patients with OUD may be administered MAT by their medical providers while they are hospitalized; this is common practice for treatment of withdrawal symptoms in the hospital but much less commonly continued upon discharge. Patients may be referred for ongoing addiction treatment as part of discharge planning.

Hospitals in the intervention condition will have three CATCH teams, each of which consists of a medical provider (MD or NP), a social worker or addiction counselor, and a peer counselor. Medical providers will be experienced addiction treatment providers who hold a waiver to prescribe buprenorphine for OUD treatment [39] and are knowledgeable about MAT. Social workers will have at least a Masters of Social Work, and addiction counselors will be eligible for NY State certification in alcohol and substance use counseling. Peers will hold a Peer Advocate certification from the New York State Office of Alcoholism and Substance Abuse Services [40]. Peer certification requires that individuals complete 46 h of training (didactic and experiential) and pass a standardized exam.

### Identification of patients with OUD

Patients can be referred for CATCH services by any member of their inpatient care team. The CATCH team will identify additional patients who may be eligible for addiction services by reviewing daily reports of current hospital inpatients having a diagnosis of OUD or opioid poisoning. Patients eligible for CATCH services are any patient with an anticipated hospital stay of at least 24 h (as determined by their medical providers) and a known or suspected substance use disorder. While CATCH teams will see patients with a range of substance use conditions, our study focuses on those with OUD.

### Inpatient services

The CATCH team will go to the bedside to meet with patients. A clinical interview by the medical provider will assess the patient’s need for and interest in receiving services, and will assess drug use and treatment history. The medical provider will make a treatment recommendation, including starting MAT as indicated. The social worker or addiction counselor will meet with the patient, on the same day if possible, to deliver motivational counseling and discuss post-discharge treatment options. The peer counselor will meet with the patient in the hospital to establish rapport, explore the patient’s motivations for treatment, and provide overdose prevention education. The peer’s role is expanded following hospital discharge, after which he/she may meet the patient in the community and escort him/her to the initial outpatient treatment visit.

### Post-discharge care

CATCH teams will strive to link patients to stable long-term MAT upon discharge. NYC has good access to MAT through its existing methadone maintenance treatment programs, and is rapidly expanding access to primary care buprenorphine treatment, and this may allow CATCH teams to rapidly connect patients to ongoing outpatient MAT. For patients who cannot be placed into care immediately, the CATCH team will staff a ‘bridge clinic’ that provides MAT on a temporary basis while continuing to work to link the patient to a stable treatment placement.

### Technical assistance

CATCH staff at each hospital will participate in a two-day in-person training prior to the launch of services. A manual will be developed that standardizes across hospitals the procedures for documenting and tracking services, initiating and titrating MAT, linking patients to treatment, and following patients post-discharge. The medical providers from all hospitals will have a bi-weekly video conference call during which they will review treatment and enrollment targets, share challenges they are encountering, and discuss patient cases. To assist with the provision of clinical care and tracking of implementation outcomes, the H + H clinical informatics department will develop a daily report, EHR documentation, and a patient registry to be used for delivering and tracking services provided by the CATCH teams.

## Data sources

Outcome measures are assessed using New York State Medicaid fee-for-service claims and managed care encounter data (referred to hereafter as *Medicaid claims and encounter data*) as the primary data source. Medicaid claims and encounter data includes primary and multiple secondary diagnoses for each hospitalization and captures all types of health care and addiction treatment visits as well as filled prescriptions that are billed to the NY Medicaid program. Data from individuals with dual eligibility for Medicaid and Medicare is not included in the Medicaid claims and encounters dataset used for analysis. The supplemental data source is the *H *+ *H clinical data warehouse* (CDW), which can be used to identify all patients (including Medicare, privately insured, and uninsured patients) who had an OUD diagnosis or received MAT during the hospital stay. The CDW captures all patients who receive addiction treatment within the H + H treatment system, which may be specialty care visits or office-based MAT prescriptions generated at any H + H facility. Overdose deaths are measured using the *DOHMH comprehensive database of NYC overdose deaths*. The DOHMH Bureau of Alcohol and Drug Use Prevention, Care, and Treatment compiles a database that links the NYC Vital Statistics Death Registry (death certificates recording cause of death) to NYC Medical Examiner Data files that document overdose deaths and all substances involved in the death [6, 41–44]. Implementation outcomes are assessed using reports that are generated on a monthly basis from EHR data, as well as qualitative interviews with CATCH staff and patients.

## Participants

There is no research recruitment of individual patients for receipt of CATCH services, which will become standard clinical care following the introduction of the intervention at each hospital. H + H policy is for all patients to sign a general HIPAA release when they first present for care, and patients will not be asked to provide separate informed consent for participation in this study; this approach is frequently used in pragmatic trials [45]. Outcomes will be measured primarily using administrative data sources. *Eligibility criteria*: Cases eligible for inclusion must meet the following criteria: adult patients (≥ 18 years), hospitalized for at least one night on an inpatient service (not including intensive care) with an admission or discharge diagnosis (based on ICD-10 codes) of opioid use disorder or opioid poisoning. *Exclusion criteria*: Received MAT in the 30 days prior to admission (as determined from Medicaid billing records).

## Study aims and outcome measures

### Primary aim

*Aim 1* is to evaluate the effectiveness of CATCH in increasing MAT initiation and engagement (primary outcomes) among patients with OUD. NYC currently has the capacity to deliver MAT to over 38,000 patients and is rapidly expanding treatment access to buprenorphine treatment in primary care [7], yet there is a large population of individuals with OUD who are not receiving treatment [13]. Treatment initiation and engagement have been selected as the key indicators of substance use quality care in the widely-used Healthcare Effectiveness Data and Information Set (HEDIS) [46, 47] and Washington Circle measures [48]. In 2018, the National Committee on Quality Assurance (NCQA) updated the treatment initiation and engagement measure to include MAT for treatment of OUD [49].

We expect that the CATCH intervention will be effective in increasing MAT initiation and engagement because it is built on a foundation of prior work demonstrating that: (1) integrating MAT with inpatient medical care is feasible and is an effective approach to engaging untreated patients in care [50]; (2) starting MAT while patients are hospitalized improves initiation and retention in outpatient treatment [25, 51] and decreases substance use; [25] and (3) providing addiction counseling and peer support during the transition from hospital to outpatient treatment increases treatment initiation and early retention [27, 52–54].

Outcome measures and data sources by specific aim are listed in Table 1. Treatment initiation is defined as having an outpatient MAT encounter within 14 days following hospital discharge. Engagement is defined as having two encounters in an outpatient MAT program or, for office-based treatment, filling two prescriptions for buprenorphine or naltrexone or receiving one prescription that covers at least 28 of the first 30 days following treatment initiation. We will also examine the total number of treatment visits in the 30-day post discharge period. The initiation and engagement measures include treatment provided through the bridge clinic, because this represents a successful transition from hospital to outpatient MAT. For patients who are discharged to another inpatient facility (e.g., skilled nursing facility), initiation will be measured from the final inpatient discharge.

**Table 1:** Outcome measures by specific aim

Specific aim	Definition	Primary data source (secondary data source)
*Aim 1 (primary aim): treatment initiation and engagement*		
Treatment initiation	Outpatient MAT encounter within 14 days of hospital discharge	Medicaid claims and encounter data (EHR data)
Treatment engagement	Receipt of 2 + additional MAT services within 30 days of initiation	Medicaid claims and encounter data (EHR data)
*Aim 2: Treatment retention*		
Rate of treatment retention	Continuous retention in treatment for 6 months	Medicaid claims and encounter data (EHR data)
*Aim 3: Acute care utilization and OD deaths*		
Acute care	Hospital and ED admissions in 6 months following discharge	Medicaid claims and encounter data (EHR data)
OD death	Poisoning death involving opioid(s)	DOHMH overdose data (Medicaid claims and encounter data)
*Aim 4: Implementation outcomes* ^a^		
Reach	Received any CATCH service(s)	EHR data
Adoption	Referrals made to CATCH by clinical staff	EHR data
Implementation fidelity	Ability to reach target population and deliver MAT	Interviews with CATCH staff and patients Monthly reports on CATCH activities
Implementation barriers and facilitators	Intervention characteristics, inner setting, outer setting, and characteristics of individuals that impact intervention delivery	Interviews with CATCH staff and patients

^a^Measured in the CATCH period only

### Secondary aims

*Aim 2* is to assess the effectiveness of CATCH in increasing retention in MAT for 6 months. Patients who remain in MAT for at least 6 months have a high probability of remaining in treatment for 12 months or longer and have better long-term treatment outcomes [55, 56]. Furthermore, patients retained in treatment for 6 months are much more likely to ‘graduate’ to long-term maintenance. [57–59].

Treatment retention is defined as receiving MAT for at least 80% of days during the 6-month period following treatment initiation (i.e., ≥ 146 days of treatment). Methadone treatment is identified by claims for each day of treatment delivered by a MMT program, and buprenorphine and naltrexone are tracked by reimbursement for filled prescriptions (including number of days of treatment prescribed). Individuals who transition between treatments (e.g., from buprenorphine to methadone) will be considered retained provided that they had at least 146 total days of MAT.

*Aim 3* is to compare the frequency of *acute care utilization* and *overdose deaths* as well as their associated costs among patients with OUD hospitalized during the CATCH period versus usual care. By effectively engaging patients with OUD in MAT, the CATCH intervention is anticipated to reduce the frequency of hospital and ED visits as well as overdose death. Decades of research on MAT, primarily on methadone treatment, demonstrate that MAT is consistently associated with less drug use and lower rates of mortality and overdose [16, 17, 60–62]. A recent meta-analysis found that overdose death rates among MAT patients are at least two-thirds lower than among individuals with OUD who left treatment [17], and MAT is a cornerstone of current efforts to reduce opioid overdose deaths in NYC [7]. Patients with untreated OUD have disproportionately high rates of acute care utilization, most of which represents preventable admissions (i.e., high-cost but low-value care) [27, 63–65]. A recent study of Medicaid patients receiving buprenorphine treatment found that the risk of any hospitalization was reduced by 18% and the risk of any ED visit was reduced by 14% among patients in treatment as compared to those who left treatment [66].

Costs for acute care admissions are frequently borne by an individual health system (e.g., costs of uninsured patients and 30-day readmission penalties), and quantifying the cost savings associated with CATCH is important for its future adoption and sustainability at H + H and in other health systems. There are also potential savings to the broader healthcare sector associated with reducing overdose deaths, including ED and hospital admissions avoided, as well as the societal value of lives saved by preventing fatal overdoses. We will compare program costs related to CATCH with savings estimates for both acute care utilization and overdose death outcomes from the perspectives of the medical care provider (H + H), the healthcare sector, and society at large.

Acute care utilization is defined as hospital and ED admissions in the 12 months following the index hospitalization. The primary measure is the total number of admissions (hospital and ED). We will also assess the number of hospital days. Overdose deaths are tracked using the DOHMH comprehensive database of NYC overdose deaths. We will identify individuals within the Medicaid database who had no claims activity in month 7 following hospital discharge and identify any matches in the overdose database. Program costs will be measured from H + H administrative records for the CATCH program that document medical and non-medical personnel assigned to the program by location.

*Aim 4* is to evaluate implementation outcomes at each CATCH hospital using a mixed-methods approach to assess the RE-AIM elements of Reach, Adoption, and Implementation fidelity. An advantage of the Hybrid Type 1 study design is that implementation measures can be collected alongside the effectiveness study for the purpose of formative evaluation; the design provides important information about the barriers to introduction and sustainability in practice [30, 31, 67]. To supplement the RE-AIM evaluation framework, we will use the Consolidated Framework for Implementation Science (CFIR) to explore in-depth the characteristics of the organization (organizational readiness, culture, priorities), individual (attitudes, norms), and intervention (complexity, relative advantage) that affect the effectiveness and implementation of the intervention. The CFIR framework incorporates theories of behavior change, and its domains include the characteristics of individuals that may lead to behavior change (e.g., knowledge and beliefs, stage of change, and self-efficacy) as well as the interplay between individuals, the context in which the intervention is provided (inner setting and outer setting), and the characteristics and delivery of the intervention itself, which may not be fully captured by RE-AIM [68, 69]. Findings will help us to interpret any differences in effectiveness across sites and populations, provide insight into potential barriers and facilitators of full scale implementation, and inform plans for future dissemination of the model to additional hospitals, both within the H + H system and in other health systems.

*Reach* is defined as the proportion of patients with OUD who receive CATCH services. A descriptive analysis will use EHR data to ascertain the proportion of patients with OUD who had at least one contact with the CATCH team during the 6-month study period. *Adoption* is the proportion of eligible patients referred to the CATCH service by clinical staff. Additionally, we will assess the characteristics (demographics, comorbidities, inpatient service (medical/surgical/psychiatric), and number of hospital days) of OUD patients who were referred versus those who were not referred. *Implementation fidelity* considers the delivery of MAT to the target population and barriers to providing high-quality care in the hospital and post-discharge. Our fidelity measures are informed by the CFIR framework and are both quantitative and qualitative. Process measures capture the planning and execution of the intervention and are primarily quantitative. These measures will be assessed quarterly for a total of 12 months after CATCH introduction at each hospital, using registry and EHR data. Qualitative interviews will characterize intervention characteristics, inner and outer setting, and characteristics of individuals that may affect the implementation and effectiveness of the intervention. Individual interviews will be conducted with CATCH staff and patients during early implementation of the program and 9–12 months post-implementation of CATCH (five staff and five patients per hospital). Patient interviews will include those who both receive and decline CATCH services. A purposive sampling approach will be used to select CATCH staff and patients with a variety of roles, demographic characteristics, and backgrounds to participate in interviews, and participants will receive $50 compensation.

## Analysis plan

Power analysis and sample size calculations: Simulations were used to assess the statistical power to detect a range of effect sizes for the probability of initiation of post-discharge MAT over a 12-month period. Based on Medicaid claims and encounter data from the participating hospitals, we expect that 7% of the individuals under treatment as usual will initiate MAT (range is 4–9%). We conservatively assume that the variance of the cluster-level random effect on the logistic scale is approximately 0.16, which is equivalent to an ICC of 0.05. In simulations, 1000 iterations were used to estimate the power. For each iteration, a generalized linear mixed model was fit to randomly generated data and p-values were assessed to determine if the effect was detected. With six hospitals having approximately 800 patients each, we have power to detect these effects using a two-sided, 0.05-level test. Specifically, we have 80%, 95%, and 99% power to detect effect sizes of 0.13, 0.18, and 0.23, respectively; these effect sizes translate to a treatment initiation rate in the intervention group of 20%, 25%, and 30%, respectively.

The analysis of the effect of the intervention on the primary and secondary outcome measures of MAT initiation, engagement, and retention, in the context of a stepped wedge cluster-randomized trial, will proceed using a Generalized Linear Mixed Model (GLMM). In particular, we will use a random effects model that assumes a binomial distribution with a logit link function. The comparison will make no linear assumption about the time trend for each outcome over the course of the trial. The model will be as follows:

1 $$ {\text{logit}}(Y_{ijt} ) = \mu + \alpha_{j} + \theta_{t} + \tau C_{i} , $$

where $Y_{ijt} = 1$ if the *i*th subject within the *j*th hospital in time period *t* initiates addiction treatment within 14 days of the index visit, Y_ijt_ = 0 otherwise. α_j_ is a random effect for hospital *j* with mean 0 and variance σ_b_^2^. θ_t_ is a time-specific effect of period t, t ϵ (1, 2,…, 12). C_i_ is an indicator variable of treatment arm; C_i_ = 1 if individual *i* receives care under the CATCH intervention, and C_i_ = 0 if individual *i* receives TAU. τ represents the treatment effect of the intervention. The model will be fit using the LME4 package using R software. The hypothesis will be tested using two-sided level of significance α = 0.05. For secondary outcomes of acute care utilization and OD deaths, we will apply GLMMs with a log link and the Poisson distribution.

Because outcome data will be derived from administrative data, and we do not anticipate having missing data. However, in the event that there is missing data, we will use multiple imputation under the assumption of data missing at random (MAR). This is consistent with following an intent-to-treat protocol. We will also assess potential confounders of treatment effect on the primary and secondary outcomes (e.g., medical or psychiatric comorbidities, non-opioid SUDs, sex, and other demographic characteristics) that may vary across the participating hospitals. Confounders will be included in the model as additional fixed effects based on change-in-estimate criterion [70, 71].

### Economic analyses

Net economic benefit will be defined as estimated savings attributable to CATCH minus the program costs of implementing CATCH. National labor rates will be assigned to personnel costs to improve generalizability [72]. We will assign savings associated with reductions in uncompensated care, 30-day readmission penalties, or length of stay to the differences observed between the CATCH and TAU conditions in hospital and emergency department (ED) admissions during the 6 months following discharge. We will follow established guidelines for conducting economic evaluations according to the recommended healthcare sector and societal perspectives [73]. From the healthcare sector perspective, the benefit of CATCH will be calculated by assigning standard costs to each category of acute care utilization (ED visit or hospitalization) based on Medicare fee schedules. Cost measures will follow standard practice using Medicare fee schedules as proxies for relevant ambulance, ED, and hospitalization costs. From the societal perspective, the benefit will include healthcare system savings plus the monetary value representing the benefit of each overdose avoided. We will include future productivity and consumption effects, health-related quality-of-life effects, and longevity effects using a threshold value of $100,000/quality-adjusted life year (QALY) for the latter two effects [74].

### Qualitative analyses

Interviews will be audio recorded and transcribed. Coding will be done by two researchers trained in Atlas.ti [75], using an a priori coding scheme based on the CFIR domains and will identify any emergent themes using a grounded theory approach. Findings will be discussed among the investigators to reach consensus on the main themes and any adaptations to the CATCH implementation strategy that might support its sustainability and future implementation at other sites.

## Discussion

By offering a model for effectively engaging hospital patients with OUD in highly effective yet underutilized treatment, the CATCH intervention has the potential to significantly improve population health. Our study utilizes a rigorous stepped-wedge study design that will generate robust and unbiased results for our primary and secondary outcomes. As a pragmatic trial, the research is conducted in collaboration with the health system in real-world settings and at scale, and, as a hybrid Type 1 study, it measures implementation outcomes simultaneously with the effectiveness study to inform future dissemination of the CATCH model.

Despite the study’s strengths, there remain some limitations. While the vast majority of OUD patients in the participating hospitals are covered by Medicaid (74% in our pilot data), we will not have comprehensive data on non-Medicaid patients. For non-Medicaid patients, we will rely on EHR data collected in the H + H clinical data warehouse to measure any care received at any of the 70 + inpatient and outpatient facilities in the H + H system. H + H is the leading provider of addiction treatment for safety net populations in NYC, and we therefore anticipate capturing many of the addiction treatment visits by the non-Medicaid population. For current inmates in the New York City jail system, inpatient hospital care is billed to Medicaid, but Medicaid coverage is suspended after they return to jail. We thus have limited ability to look at post-discharge treatment in the inmate population using Medicaid claims and encounter data. However, because H + H is the provider of medical and psychiatric care in the New York City jails, their treatment (including MAT) is captured in the correctional health services EHR, which we may be able to use to assess post-discharge treatment.

There may be under-coding of OUD in billing data and medical records [76–79]. To assess this, we will conduct a structured chart review at each participating hospital of the medical records for 100 patients who received MAT in the hospital to determine whether these patients have documented OUD. We will compare the chart review findings to the ICD-10 diagnostic codes used for the hospitalization to determine the frequency with which OUD is not captured in diagnostic coding. If fewer than 90% of patients identified in the chart review have an ICD-10 discharge diagnosis of OUD or poisoning, we will revise our process for identifying cases eligible for CATCH services.

Finally, while non-fatal OD is even more common than OD death, there is no reliable measure of this outcome in existing administrative data. This study is conducted in an urban public hospital system, and our findings will not necessarily generalize to other settings. However, health systems like H + H are the most affected by the opioid epidemic, and our findings will be relevant to the management of high-risk and high-cost populations in similar systems across the US.

In summary, by transforming hospitalization into an opportunity to engage patients with OUD in highly effective yet underutilized treatment, addiction consult services have the potential to play a significant role in addressing the opioid crisis—but rigorous study is needed to support the adoption and value of this approach. This study will provide the first evaluation of an addiction consult model in a multi-site trial and promises to generate knowledge that can rapidly transform practice and inform the intervention’s potential for dissemination and sustainability.

## Abbreviations

OUD: opioid use disorder
OD: drug overdose
H + H: NYC Health and Hospitals system
DOHMH: Department of Health and Mental Hygiene
CATCH: Consult for Addiction Treatment and Care in Hospitals
MAT: medication for addiction treatment
TAU: treatment as usual
SUD: substance use disorder
EHR: electronic health record
GLMM: generalized linear mixed model
MAR: missing at random
ED: emergency department
QALY: quality-adjusted life year
